# Association Study Between Methylation in the Promoter Regions of cGAS, MAVS, and TRAF3 Genes and the Risk of Cervical Precancerous Lesions and Cervical Cancer in a Southern Chinese Population

**DOI:** 10.3389/fgene.2019.01123

**Published:** 2019-11-14

**Authors:** Shiqi Huang, Ruixin Li, Xiuxia Huang, Shaoling Zheng, Lijun Wang, Zihao Wen, Xiaoqian Zou, Jing Wu, Yumei Liu, Dandan Liu, Yao Wang, Shirui Dong, Xiaojing Chen, Kehui Zhu, Xiuben Du, Zixing Zhou, Yajing Han, Xiaohong Ye, Chengli Zeng, Baohuan Zhang, Guang Yang, Chunxia Jing

**Affiliations:** ^1^Department of Epidemiology, School of Medicine, Jinan University, Guangzhou, China; ^2^Department of Gynecologic Oncology, Sun Yat-sen Memorial Hospital, Sun Yat-sen University, Guangzhou, China; ^3^Department of Nutriology, School of Medicine, Jinan University, Guangzhou, China; ^4^Department of Pathogen Biology, School of Medicine, Jinan University, Guangzhou, China; ^5^Guangdong Key Laboratory of Environmental Pollution and Health, Jinan University, Guangzhou, China

**Keywords:** cervical precancerous lesions, cervical cancer, *cGAS*, *MAVS*, *TRAF3*, gene promoter methylation, interaction

## Abstract

A case-control study was used to explore the association between the methylation status in the promoter regions of the *cGAS*, *MAVS*, and *TRAF3* genes and the diseases of cervical precancerous lesions (CPL) and cervical cancer (CC) in a Southern Chinese population, and to further explore their interaction effects with high-risk human papillomavirus (hrHPV) infection and environmental factors in these diseases. The study protocol was approved by the ethics committee of The First Affiliated Hospital of Jinan University, and this study was performed in 97 healthy controls, 75 patients with CPL and 33 patients with CC, while each participant has read and signed the informed consent forms before enrolment. The promoter methylation status genes were detected from the bisulfite-treated DNA by the bisulfite sequencing PCR (BSP) technique, which was carried out using MethPrimer. The *cGAS*, *MAVS*, and *TRAF3* promoter methylation levels in CPL (CPL*_cGAS_* = 35.40%, CPL*_MAVS_* = 24.26%, and CPL*_TRAF3_* = 96.76%) were significantly higher than those in the control (Control*_cGAS_* = 31.87%, Control*_MAVS_* = 21.16%, and Control*_TRAF3_* = 96.26%, *P_cGAS_*< 0.001, *P_MAVS_*< 0.001, and *P_TRAF3_* = 0.001); however, there was no significant differences between the CC and control. In the logistic regression model with adjusted covariates, compared with the individuals whose *cGAS* methylation levels were less than or equal to 31.87%, the women with the levels more than 31.87% increased the risk of CPL by 2.49 times (OR^a^ = 2.49, 95% CI = 1.31-4.75, *P*^a^ = 0.006). The women with *MAVS* methylation levels above 21.16% were 1.97 times more likely to have CPL than the those with the levels less than 21.16% (OR^a^ = 1.97, 95% CI = 1.06–3.69, *P*^a^ = 0.033). A synergistic interaction was found between hrHPV and gene promoter methylation levels of *cGAS* and *MAVS* in CPL; however, no potential interaction was observed in CC. The promoter methylation levels in *cGAS*, *MAVS*, and *TRAF3* genes are higher in CPL than in control, indicating that hypermethylation might be an early event in the progression of cervical intraepithelial neoplasia (CIN). The interaction between the promoter methylation levels in *cGAS* and *MAVS* genes and hrHPV infection might play a role in the development of CPL.

## Introduction

Abnormal DNA methylation in the gene promoter region is a well-recognized epigenetic hallmark in the premalignant and malignant stages of cancers, and it has been observed in different kinds of genes, such as tumor suppressor genes and DNA repair genes (Merlo et al., 1995; Esteller et al., 1999a; Esteller et al., 2000; Guo et al., 2009; Zou et al., 2009; Esteller et al., 1999b). Methylation of the CpG sites in promoter regions can lead to gene dysfunction or inactivation, causing tumor progression (Nojima et al., 2001; Ribeiro-Filho et al., 2002). Several studies have reported that promoter methylation status of multiple genes is associated with cervical precancerous lesions (CPL) and cervical cancer (CC) development, such as CDH13, CDKN2B, TIM3, and RASSF1A (Dong et al., 2001; Missaoui et al., 2011a; Li et al., 2015a). Methylation has been detected at cervical precancerous stages, and methylated DNA is a relatively stable target and allows for flexibility of assay development, suggesting that methylation markers may have value in cervical diseases screening (Wentzensen et al., 2009; Steenbergen et al., 2014). DNA methylation of promoter regions is associated with the cervical disease, but very few studies have been reported for gene promoter methylation related to antiviral innate immunity pathways, moreover, the research evaluating DNA methylation and epidemiologic factors for CPL and CC is very limited in Chinese women.

CC is one of the most common malignancies in women, with an estimated 530,000 new cases and 275,000 deaths worldwide per year (Zhao et al., 2012). Approximately 90% of the deaths from CC occurred in low- and middle-income countries during 2015. The mortality of CC has significantly decreased in China, but a younger age trend and a substantial increase in CC incidence have been seen in some regions (Shi et al., 2012; Chen et al., 2016). The incidence and mortality of cervical disease vary widely among different populations, geographic areas and time periods in China (Li et al., 2011). The incidence and mortality rates of CC could be reduced through effective screening, early prevention and diagnosis (Peto et al., 2004b). High-risk human papillomavirus (hrHPV) is the cause of CPL and CC (Mj et al., 2005), and hrHPV prevalence is well correlated with the risk of diseases, particularly in middle-age women (Peto et al., 2004a; Maucort-Boulch et al., 2008; Bouvard et al., 2009). The squamous intraepithelial lesions (SILs) of the cervix are referred to as CPL, which can be divided into low-grade SIL (LSIL or cervical intraepithelial neoplasia 1, CIN1) and high-grade SIL (HSIL or CIN2/3) histologically. The progression from LSIL through HSIL and eventually to invasive cancer lasts up to 10–30 years (Winer et al., 2005; Lendvai et al., 2012). The periods of CPL are generally chronic and reversible; therefore, various methods can be adopted to prevent carcinogenesis, and the stage of CPL might be affected by many genetic or environmental factors (Armstrong and Doll, 1975).

Viral infection of the host can trigger innate immune responses, and previous studies have revealed that cyclic GMP-AMP synthase (*cGAS*)/*STING* and *RIG-I*/*MAVS* pathways play important roles in host innate immunity against HPV-induced precursor lesions and invasive cancer of the uterine cervix (Karim et al., 2013; Lau et al., 2015; Xiao et al., 2016). *cGAS*, also known as MB21D1 or C6orf150, is identified as a general cytosolic DNA sensor, which can detect intracellular DNA and catalyze the synthesis of second messenger cyclic-GMP-AMP (cGAMP) from ATP and GTP (Sun et al., 2013). Then synthesized cGAMP binds to and activates the adaptor protein *STING* (stimulator of interferon genes) to induce the phosphorylation of downstream factors, including TANK binding kinase 1 (*TBK1*) and interferon regulatory factor 3 (*IRF3*), thereby triggering type-I interferon (IFN) production (Wu et al., 2013; Lau et al., 2015). Type I IFNs are critical for antiviral autoimmune responses. The *RIG-I*/*MAVS* pathway can also mediate IFN production in response to cytosolic double-stranded RNA or single-stranded RNA containing 5’-triphosphate (5’-ppp) (Kawai et al., 2005; Hornung et al., 2006). RNA polymerase III (Pol-III) has been suggested to function as a potential DNA sensor that can identify and convert cytosolic DNA into 5’-triphosphate (5’-ppp) RNA (Hornung et al., 2006; Pichlmair et al., 2006; Chiu et al., 2009), which can be detected by *RIG-I* sensor and lead to the mitochondrial assembly of mitochondrial antiviral-signaling (*MAVS*) complexes. Then, *MAVS* binds to tumor necrosis factor receptor-associated factor 3 (*TRAF3*) and recruits TBK1/IKKε kinases that activate NF-κB and direct the phosphorylation of constitutively expressed IRF3 to induce IFN-β (Fitzgerald et al., 2003; Oganesyan et al., 2006; Hiscott, 2007; Matthys et al., 2014).

In this study, we investigated the promoter methylation status of *cGAS*, *MAVS*, and *TRAF3* in CPL and CC, and we further explored the association between potential biological interactions and the risk of CPL and CC in the Southern Chinese population.

## Materials and Methods

### Study Participants

A total of 205 Southern Chinese women were recruited in our study, including 97 healthy women in the control group and 108 patients with CPL and CC. The mean age was 45.50 years, ranging from 20 to 65 years. The classifications of the groups were according to the ThinPrep cytologic test (TCT) with the Bethesda system for reporting cervical cytology (TBS 2001) and cervical biopsies were confirmed by two pathologists. The women in the control group were negative for CPL or CC without other diseases of uterus or cervix. The CPL group (n = 75) was women with SILs, including 38 with LSILs and 37 with HSILs. There were 33 patients in the CC group.

Epidemiological data were obtained from the face-to-face questionnaire of each study subject; these questionnaires were conducted by qualified investigators and a doctor to ensure the accuracy and authenticity of the information. In addition, peripheral venous blood and cervical epithelial exfoliated cells were also collected from each participant. The blood sample was gathered with EDTA vacuum collection tubes and maintained at 4 degrees; then, blood samples were transported to our laboratory for DNA and RNA extraction. Cervical epithelial exfoliated cells were collected by cytobrush (QIAGEN, Valencia, CA) and preserved in denaturation buffer. ThinPrep 2000 (Hologic Inc.) and SurePath liquid-based Pap test (BD, USA) were used for the TCT. HPV DNA from cervical samples was extracted from the commercial magnetic beads kit (Chemagen, PerkinElmer, Waltham, MA), which was performed in the clinical standard laboratory of BGI (Beijing Genomics Institute, Shenzhen, China). Both hrHPV (types 16, 18, 31, 33, 35, 39, 45, 51, 52, 56, 58, 59, 66, and 68) and low-risk HPV (lrHPV, types 6 and 11) were detected with MassARRAY (Sequenom, Sandiego, CA) based on the matrix-assisted laser desorption/ionization time of flight (MALDI-TOF) mass spectrometry (MS).

### Methylation Status Detection

**Figure 1:**
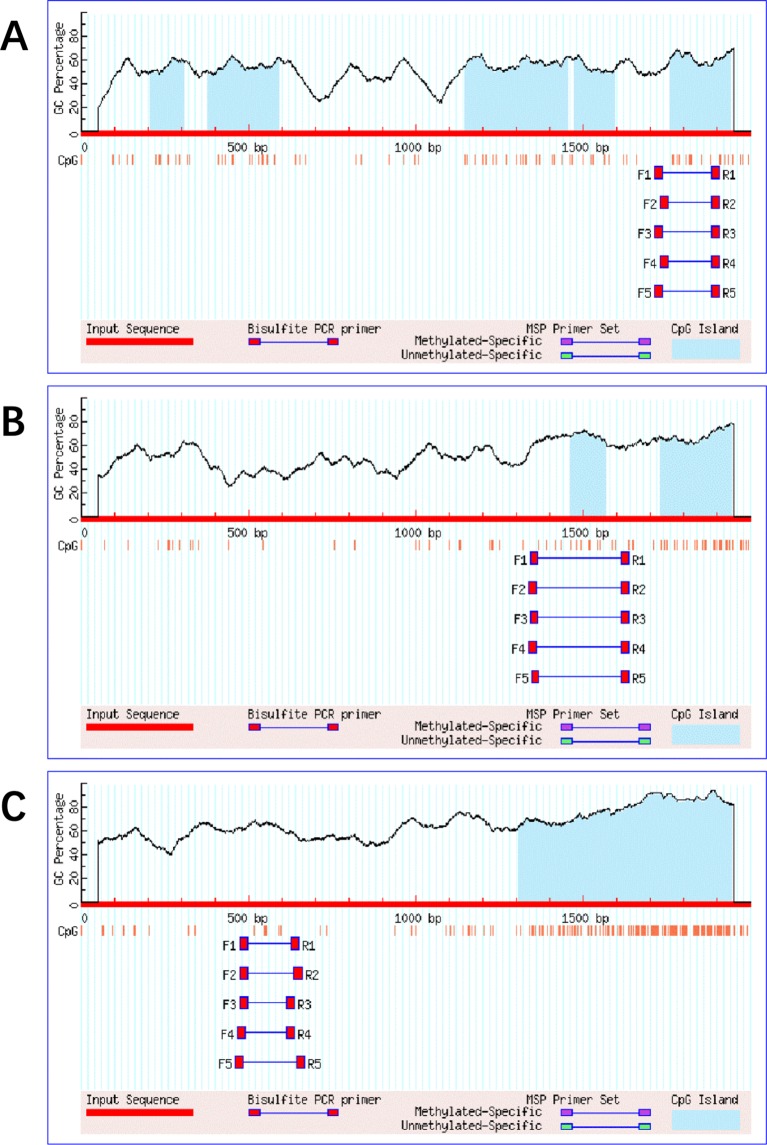
The target sequences' bisulfite transformation and design primers in the promoter regions of genes cGAS/MAVS/TRAF3.

The promoter region is approximately 2000-bp length upstream of the gene transcription start site, and the reference promoter sequences for the human *cGAS, MAVS*, and *TRAF3* genes are obtained from the UCSC Gene Sorter (http://www.genome.ucsc.edu/cgi-bin/hgNear). The methylation status is described as percentage of methylated CpGs of the possible CpG methylation sites, which is detected by the bisulfite sequencing PCR (BSP) technique. The BSP is the most widely used method to give information about the methylation profile of every single CpG site in a given sequence. We use MethPrimer (http://www.urogene.org/methprimer/index1.html) to carry out the bisulfite transformation of the target sequences and design primers (Li and Dahiya, 2002), which is based on key CpG island prediction in the promoter regions of genes *cGAS*/*MAVS*/*TRAF3* (**Figure 1**).

Blood DNA was extracted using the QIAamp DNA Mini Kit (QIAGEN, 51104), and the bisulfite-treated DNA was purified using the Wizard DNA Clean-up System (Promega, A7290) after DNA denaturation and bisulfite conversion. The DNA was PCR-amplified by using the EpiTaq HS Kit (TaKaRa, R110A) in a 20-µl reaction volume with the designed primers (**Table S1**). The PCR conditions consisted of an initial incubation for 3 min at 98°C, followed by 40 cycles of 10 s at 98°C, 30 s at 55°C, and 30 s at 72°C, and finally 10 min at 74°C. The PCR product was sequenced by IGE (Guangzhou IGE Biotechnology, LTD., China), and the sequencing results were analyzed using Chromas software. There were 9, 10, and 5 CpG sites detected in the promoter region of the *cGAS*, *MAVS*, and *TRAF3* genes, respectively. The methylation status was dependent on the peak height of cytosine (C) and thymine (T) signals according to the following formula:

$$\begin{array}{l} \text{The}\;\text{methylation}\;\text{rate}\;\text{of}\;\text{single}\;\text{CpG}\;\text{site}\;(\%)\;\\ \text{= }\frac{\text{Peak height of C}}{\text{Peak height of C + Peak height of T}}\times \text{100\% } \end{array}$$

$$\text{The methylation level (\%) = }\frac{\text{Methylation rate of total CpG sites}}{\text{Number of CpG sites}}$$

### Statistical Analyses

The demographic and experimental data were obtained and recorded, and the database was established by Epidata software. Data analyses were performed with SPSS software (version 24.0, Inc., Chicago, USA). Differences among the groups of control, CPL, and CC were assessed by analysis of variance (ANOVA), Dunnett’s t-test, chi-squared test, and Mann-Whitney U test. Correlation analysis was conducted using the logistic regression model, which was calculated by estimating the odds ratios (ORs) and 95% confidence intervals (95% CIs) after adjusting for potential covariates. Potential two-factor interaction was evaluated on an additive scale with a 95% CI by calculating the following three measures: synergy index (S), attributable proportion due to interaction (AP), and relative excess risk due to interaction (RERI) (Skrondal, 2003; Andersson et al., 2005). If there were no biological interactions, then the 95% CI of S is over 1, and RERI and AP are cross 0 (Assmann et al., 1996; Andersson et al., 2005). Multifactor dimensionality reduction software 1.0.0 (MDR 1.0.0) was used to explore the multifactor interactions, and the best model was determined by the testing balanced accuracy (TBA) and cross-validation consistency (CVC) indexes. All *p* values below 0.05 were considered statistically significant.

## Result

### Demographic Characteristics and Relevant Factors Analysis

Demographic characteristics and analysis of relevant factors are shown in **Table 1**. The patients in the CC group were older than in the control group, and the number of pregnancies was higher in the CC group than in the control group (*p* <0.05). The positive rates of hrHPV in both the CPL (74.67%) and CC (82.35%) groups were significantly higher than in the control group (53.61%). However, there were no significant differences in other factors among the groups (*P* > 0.05).

**Table 1:** Demographic characteristics in control, CPL, and CC.

Variables	Control N = 97	CPL N = 75	CC N = 33	F/χ^2^	*p* value
Age	44.24 ± 7.14^b^	44.52 ± 7.35^c^	51.61 ± 9.88^b^	12.212	**<0.001**
BMI	22.02 ± 3.31	22.06 ± 2.55	22.43 ± 3.05	0.241	0.786
Age at menarche	15.21 ± 1.74	15.47 ± 2.64	14.32 ± 1.54	3.047	0.050
Age at first intercourse	22.47 ± 3.97	22.63 ± 3.18	22.63 ± 2.91	0.048	0.953
Age at primiparity	23.81 ± 3.40	23.88 ± 3.58	23.06 ± 5.36	0.551	0.577
Number of pregnancies	3.02 ± 1.28^b^	3.25 ± 1.39 ^c^	3.72 ± 1.63 ^b^	3.141	**0.045**
Number of births	2.38 ± 0.93	2.67 ± 1.78	2.69 ± 1.47	1.725	0.181
Number of abortions	0.64 ± 0.88	0.59 ± 0.95	1.03 ± 1.36	2.423	0.091
HrHPV infection					
Negative	45 (46.39)	19 (25.33)	3 (17.65)	10.784	**0.005**
Positive	52 (53.61)^b^	56 (74.67)^b^	14 (82.35)^b^		
Unavailable	0	0	16		
Passive smoking					
Negative	38 (39.20)	24 (32.00)	12 (41.40)	1.24	0.538
Positive	59 (60.80)	51 (68.00)	17 (58.60)		
Unavailable	0	0	4		
Physical exercise					
Negative	84 (86.60)	67 (89.30)	25 (75.76)	3.562	0.169
Positive	13 (13.40)	8 (10.70)	8 (24.24)		

Data is shown means ± standard deviation for continuous variable, and the number of samples is shown for categorical variable. P value is based on ANOVA or Chi-square test. Bold values indicate the statistically significant p value (p < 0.05). The same letter of b/c indicates that the significant differences compared with the control group. CPL, cervical precancerous lesions; CC, cervical cancer; HrHPV, high-risk HPV; BMI, body mass index, kg/m^2^. HrHPV positive is including HPV negative and low-risk HPV positive.

### Gene Promoter Methylation Status in Control, CPL, and CC

**Figure 2:**
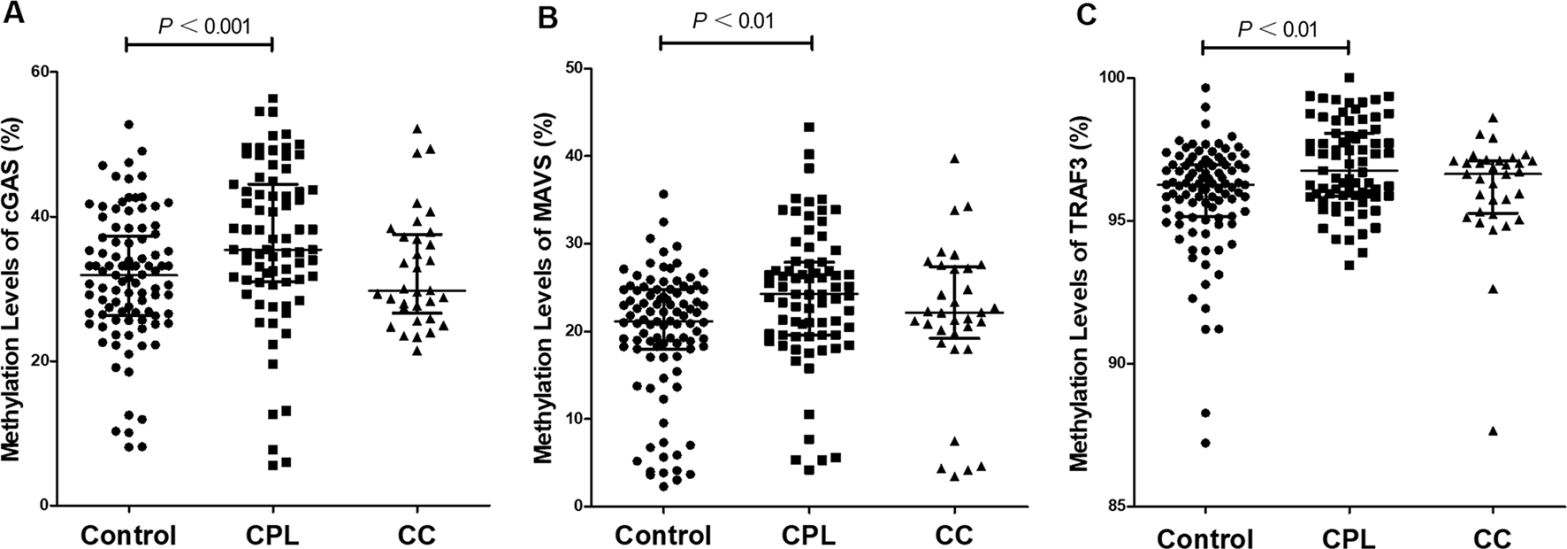
Comparison of gene promoter methylation levels. **(A)** Cyclic GMP-AMP synthase (*cGAS*), **(B)** mitochondrial antiviral-signaling (*MAVS*), **(C)** tumor necrosis factor receptor-associated factor 3 (*TRAF3*). The reference is control group, and differences between pairs were assessed using Mann-Whitney U test. Scatter dot plot represents the methylation level and the line in plot present the median with interquartile range.

The methylation rate of a single CpG site and the methylation levels of gene promoter regions are presented in **Table 2**. The methylation status of the single CpG site was as follows: the methylation level of the *cGAS*, *MAVS*, and *TRAF3* genes and the methylation rates of the C1-C8 CpG sites of *cGAS*, M1-M9 CpG sites of *MAVS*, and T1-T4 CpG sites of *TRAF3* were higher in the CPL group than in the control group (*P* < 0.05); C1 and C2 in *cGAS*, M4 and M10 in *MAVS*, as well as T3 in *TRAF3* were higher in the CC group than in the control group (*P* < 0.05). The methylation levels of the *cGAS*, *MAVS*, and *TRAF3* gene promoter regions in the CPL group (CPL*_cGAS_* = 35.40%, CPL*_MAVS_* = 24.26%, and CPL*_TRAF3_* = 96.76%) were significantly higher than in the control group (Control*_cGAS_* = 31.87%, Control*_MAVS_* = 21.16%, and Control*_TRAF3_* = 96.26%; *P_cGAS_*< 0.001, *P_MAVS_*< 0.001, and *P_TRAF3_* = 0.001), while there were no significant differences between the CC and control groups (**Figure 2**).

**Table 2:** Promoter methylation status of cyclic GMP-AMP synthase (*cGAS)*/mitochondrial antiviral-signaling (*MAVS*)/tumor necrosis factor receptor-associated factor 3 (*TRAF3*) in control, cervical precancerous lesion (CPL), and cervical cancer (CC).

Methylation status	Median (%)			*p* value	
	Control	CPL	CC	Control/CPL	Control/CC
CpG site of *cGAS*					
C1	62.09	66.29	67.38	**0.001**	**0.003**
C2	50.14	54.24	53.95	**0.005**	**0.012**
C3	24.37	30.50	19.57	**0.001**	0.238
C4	25.51	28.00	23.47	**0.036**	0.837
C5	29.87	38.37	29.00	**<0.001**	0.904
C6	31.98	40.17	31.58	**0.002**	0.523
C7	32.33	38.65	29.81	**<0.001**	0.673
C8	19.11	26.24	17.29	**<0.001**	0.554
C9	8.57	9.00	6.18	0.477	0.056
Methylation level of *cGAS*	31.87	35.40	29.75	**<0.001**	0.925
CpG site of *MAVS*					
M1	12.46	15.46	12.67	**0.005**	0.808
M2	5.70	7.93	7.26	**0.003**	0.121
M3	8.41	10.34	10.79	**0.019**	0.165
M4	5.09	6.93	6.79	**0.002**	**0.026**
M5	23.87	28.07	24.69	**0.003**	0.415
M6	54.19	56.70	55.07	**0.005**	0.313
M7	51.73	53.62	53.01	**0.005**	0.242
M8	24.32	26.92	23.30	**0.042**	0.433
M9	12.25	16.12	14.24	**0.007**	0.462
M10	6.58	6.98	9.53	0.160	**0.032**
Methylation level of *MAVS*	21.16	24.26	22.12	**<0.001**	0.236
CpG site of *TRAF3*					
T1	95.48	95.96	95.71	**0.013**	0.606
T2	98.12	100.00	100.00	**0.021**	0.079
T3	97.21	100.00	100.00	**0.006**	**0.006**
T4	97.11	97.98	96.73	**0.019**	0.206
T5	93.03	93.65	92.76	0.227	**0.047**
Methylation level of *TRAF3*	96.26	96.76	96.65	**0.001**	0.324

Statistically significant p values (p < 0.05) in bold font were based on the Mann-Whitney U test.

### Association of the Gene Promoter Methylation Status With the Risk of CPL and CC

We investigated the relationship between the gene promoter methylation status of *cGAS*, *MAVS*, and *TRAF3* and the diseases of CPL and CC. The methylation levels in both *cGAS* and *MAVS* were associated with CPL after adjusting for age and number of pregnancies, but no associations were observed between the methylation levels and CC group (**Table 3**). Compared with the individuals whose methylation levels in *cGAS* were less than or equal to 31.87%, the other individuals had an increased risk of CPL by 2.49 times (OR^a^ = 2.49, 95% CI = 1.31–4.75, *P*^a^ = 0.006). Moreover, individuals with *MAVS* methylation levels above 21.16% had a 1.97 times higher risk of CPL than the others (OR^a^ = 1.97, 95% CI = 1.06–3.69, *P*^a^ = 0.033). After adjusting for age and number of pregnancies, C1, C2, C3, C5, C6, C7, and C8 of *cGAS*, M2, M3, M5, M6, M7, and M9 of *MAVS* and T2, T3, T4, and T5 of *TRAF3* were associated with CPL, and C1, C2, C8, and C9 of *cGAS* were associated with the CC group (**Table S2**).

**Table 3:** Association analysis of the promoter methylation level of cyclic GMP-AMP synthase (*cGAS*)/mitochondrial antiviral-signaling (*MAVS*)/tumor necrosis factor receptor-associated factor 3 (*TRAF3*) gene with the risk on cervical precancerous lesion (CPL), and cervical cancer (CC).

Group	Methylation level		OR (95% CI)	*P*	OR^a^ (95% CI)	*P*^a^
	Low	High				
*cGAS*						
Control	49 (50.52)	48 (49.48)	1.00		1.00	
CPL	23 (30.67)	52 (69.33)	2.31 (1.23–4.34)	**0.009**	2.49 (1.31–4.75)	**0.006**
CC	18 (54.55)	15 (45.45)	0.85 (0.39–1.88)	0.689	0.91 (0.38–2.19)	0.832
*MAVS*						
Control	49 (50.52)	48 (49.48)				**`**
CPL	26 (34.67)	49 (65.33)	1.92 (1.04–3.58)	**0.039**	1.97 (1.06–3.69)	**0.033**
CC	13 (39.39)	20 (60.61)	1.57 (0.70–3.51)	0.271	1.48 (0.62–3.52)	0.380
*TRAF3*						
Control	49 (50.52)	48 (49.48)				
CPL	31 (41.33)	44 (58.67)	1.45 (0.79–2.66)	0.232	1.45 (0.79–2.66)	0.237
CC	13 (39.39)	20 (60.61)	1.57 (0.70–3.51)	0.271	2.21 (0.90–5.43)	0.083

Low, cGAS ≦ 31.87%, MAVS ≦ 21.16%, TRAF3 ≦ 96.26%; High, cGAS > 31.87%, MAVS > 21.16%, TRAF3 > 96.26%. Statistically significant p values (p < 0.05) in bold font was based on logistic regression. ^a^Adjusted odds ratio (OR) on age and number of pregnancies.

### Association between the Biological Interaction and the Diseases of CPL and CC

There were no relationships between hrHPV and gene promoter methylation levels in the different groups (**Table S3**); therefore, we further explored the biological interactions of hrHPV and methylation levels and the estimated risk of CPL and CC. A synergistic interaction was found between the presence of hrHPV and the gene promoter methylation levels in *cGAS* as well as in *MAVS* in CPL (AP^a^*_cGAS_* = 0.54, 95% CI = 0.13–0.95; AP^a^*_MAVS_* = 0.50, 95% CI = 0.01–0.99), and no potential interactions were observed in CC (**Table S4**).

**Figure 3:**
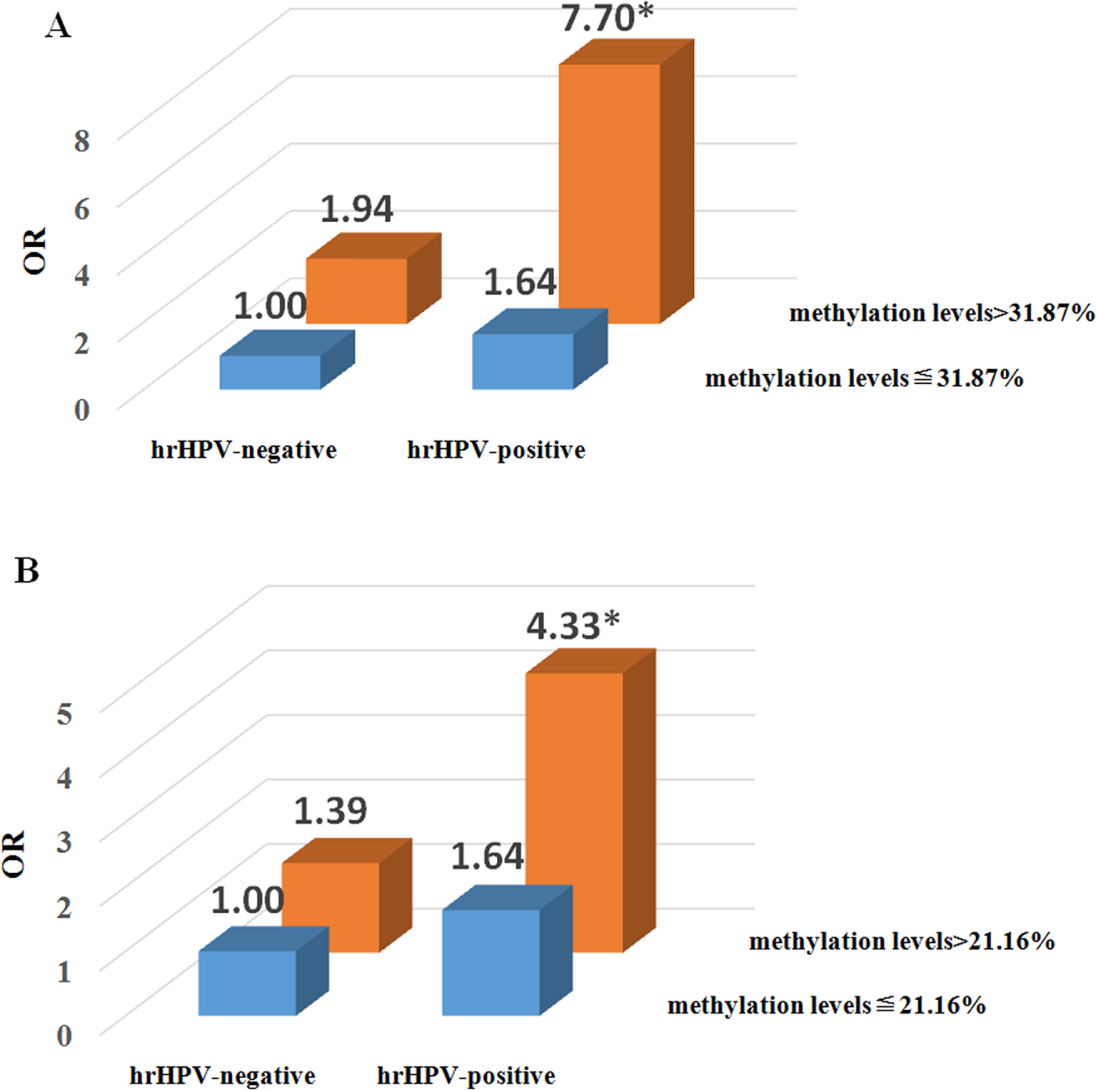
Risk analysis of the two-factor interaction of high-risk human papillomavirus (hrHPV) and promoter methylation levels in the cervical precancerous lesion (CPL). **(A)** The interaction between hrHPV and methylation level of cyclic GMP-AMP synthase (*cGAS*). The reference group is the combination of hrHPV-negative (including lrHPV-positive and HPV negative individuals) and *cGAS* methylation levels ≦ 31.87%. *P 0.001, adjusted odds ratio (OR) on age and number of pregnancies. **(B)** The interaction between hrHPV and methylation level of mitochondrial antiviral-signaling (*MAVS*). The reference group is the combination of hrHPV-negative (including lrHPV-positive and HPV negative individuals) and *MAVS* methylation levels ≦ 21.16%. *P = 0.001, adjusted OR on age and number of pregnancies.

The interactions of the presence of hrHPV and methylation levels of *cGAS* and *MAVS* were associated with an increased risk of CPL (**Tables S5** and **S6**). As shown in **Figure 3A**, the individuals with hrHPV infection and *cGAS* methylation levels above 31.87% might increase the 7.70-fold risk of CPL compared with the others, who had *cGAS* methylation levels lower than or equal to 31.87% and were hrHPV-negative (OR^a^ = 7.70, 95% CI = 2.84-20.88, *P*^a^ < 0.001). Moreover, compared with the individuals who had *MAVS* methylation levels less than or equal to 21.16% without hrHPV infection, the risk of CPL was 4.33-fold higher in those who were hrHPV-positive and had *MAVS* methylation levels above 21.16% (OR^a=^ 4.33, 95% CI = 1.78-10.52, *P*^a^ = 0.001) (**Figure 3B**).

The MDR analysis was used to test the interaction among gene promoter methylation levels, hrHPV infection and some other variables; however, multifactor interaction models were not found in CPL or CC (**Table S7**).

## Discussion

This is the first study that describes the roles of DNA methylation in the promoter regions of the important innate immune pathways of *cGAS*, *MAVS*, and *TRAF3* in CPL and CC. The methylation levels in the promoter regions of *cGAS*, *MAVS*, and *TRAF3* were significantly increased in the CPL group compared with the healthy control group, while there were no statistically significant differences between the control and CC groups, suggesting that aberrant DNA methylation in the gene promoter regions of *cGAS*, *MAVS*, and *TRAF3* are the early events in the progression of cervical neoplasm.

The carcinogenesis of cervix exhibits the dynamics of DNA methylation changes (Widschwendter et al., 2007; Zhuang et al., 2012; Xia L, 2016), which remains largely unexplored. DNA methylation alterations across the numerous genes are seen even in early carcinogenesis. The cervical cancer model showed that gain of abnormal methylation at some genes in stem cells can be detected up to 3 years in advance of the appearance of precancerous cells, while this process of methylation status was changing during cancer progression (Zhuang et al., 2012). The methylation status might be affected by different factors from the stages of SIL to invasive cancer because the progression may result from the accumulation of genetic and epigenetic alterations with various environmental risks over a long period of time (Armstrong and Doll, 1975; Sadikovic et al., 2008; Szalmás and Kónya, 2009). Increasing evidence has shown that epigenetic changes as a result of aberrant hypermethylation of CpG islands in promoters or histone modifications are essential to carcinogenesis and metastasis (Saavedra et al., 2012). Our study systematically analyzed the promoter methylation status of *cGAS*, *MAVS*, and *TRAF3* genes, which can be identified as new promising methylation markers for the detection of cervical precancerous disease in the early stages.

The patient age and number of pregnancies were significantly higher in the CC group than in the control group, and no significant differences were observed between the CPL and control groups, which was consistent with the results from a study by Castle et al. that showed multiple pregnancies had little or no impact on the development of CPL (Castle et al., 2010). The association between the number of pregnancies and CC risk was also identified in Muñoz’s study (Munoz et al., 2002). Some studies revealed that older age was a risk factor for CPL and CC diseases (Netsanet et al., 2016; Teame et al., 2018). The women over 40 years old were at greater risk for invasive CC than those less than 40 years old, and this result could account for the longer period for potential exposure to the HPV virus and the time required for precancerous lesions to develop into invasive cancer (Netsanet et al., 2016). In our study, age was significantly higher only in the CC group than in the control group; this result was similar to that in the Gessesse et al. study, which documented the absence of statistically significant age in CPL (Gessesse et al., 2015).

We found that the elevated methylation levels in *cGAS* and *MAVS* promoter regions were risk factors for cervical precancerous disease after adjusting for age and number of pregnancies. The *cGAS*/*STING* and *RIG-I*/*MAVS* pathways were closely related to HPV infection and to CPL and CC (Lau et al., 2015, Xiao et al., 2016, Hajek et al., 2017), but the mechanisms are still unclear. In our study, the promoter methylation levels of *cGAS* and *MAVS* were elevated, which may affect the function of innate immunity of the *cGAS*/*STING* and *RIG-I*/*MAVS* pathways in the development of CPL. The innate immune responses are acute and temporary in the early stage, and it takes many years or even decades to advance from precancer to invasive cancer in the cervix during persistent HPV infection in women (Winer et al., 2005). Thus, the methylation levels of *cGAS* and *MAVS* might gradually become stable during the long progression of CC, with little or no impact on this stage. Abnormal methylation levels of many genes, such as tumor suppressor genes and DNA repair genes, are closely related to CPL and CC diseases (Dong et al., 2001; Feng et al., 2005; Henken et al., 2007; Jeong et al., 2010; Missaoui et al., 2011b; Li et al., 2015a), and several studies demonstrated that methylation analysis is a potential diagnostic tool for cervical precancerous or cancer screening that may potentially be used alone or in conjunction with cytology and/or HPV(Feng et al., 2005, Missaoui et al., 2011b). Moreover, a study by Bierkens indicated that methylation analysis was capable of accurately detecting HSIL that was missed by cytology, which potentially resulted from the absence of intact indicator cells or cytological sampling errors (Bierkens et al., 2013). Therefore, the aberrant methylation levels of the *cGAS* and *MAVS* gene promoters may be an early event in carcinogenesis and could act as potential markers for screening CPL at early stages.

Markedly, we found two synergistic interactions in CPL, which were hrHPV infection with *cGAS* methylation levels and hrHPV infection with *MAVS* methylation levels. DNA methyltransferases (DNMTs) are mammalian enzymes responsible for maintaining CpG methylation, and DNMT1 can be activated by both E6 and E7 of hrHPV (Verlaat et al., 2018). E6 can upregulate DNMT1 *via* p53, and E7 can directly bind to and activate DNMT1 (Burgers et al., 2007; Au Yeung et al., 2010). Conversely, silencing of E6 and E7 could reduce DNA methylation levels and restore the transformed phenotype in CC cells (Rampias et al., 2009; Li et al., 2015b; Verlaat et al., 2018). We hypothesize that DNMT1 is activated by hrHPV that methylation levels in the *cGAS* and *MAVS* promoter regions are elevated, and that there are synergistic interactions between the increased *cGAS* and *MAVS* methylation levels and hrHPV. Thus, women with hrHPV infection and higher promoter methylation levels in the *cGAS* and *MAVS* promoter region might have a higher probability of developing CPL than the others who have lower methylation levels and are hrHPV-negative. In the MDR analysis, we did not identify any gene-gene or gene-environment interaction in CPL or CC, but we cannot rule out the possibility of multiple interactions that have no roles in these diseases. This result may be due to the few environmental factors included in our analysis, so we should collect more demographic data in future research studies.

There are some limitations in our study. First, the sample size was not large enough, among which the number of cases in the CC group was small; this limitation may give rise to the increase in statistical error about probability of category II, leading to test power decrease and being prone to false-negative results. Therefore, more samples need to be collected for subsequent studies to validate our research. Additionally, the case-control study design may affect the accuracy of the methylation analysis results because of the detection delay times; therefore, we should carry a prospective observational study to dynamically monitor methylation levels at different time points and stages in future studies.

In conclusion, the methylation levels in the promoter regions of *cGAS*, *MAVS*, and *TRAF3* are higher in the CPL group than in the CC and control group, indicating that hypermethylation in the innate immunity pathway may be an early event in the progression of CIN. The methylation levels in the promoter regions of *cGAS* and *MAVS* are related to CPL; moreover, the respective interactions of hrHPV infection with *cGAS* or *MAVS* methylation levels may have roles in CPL. Our study provides a new epidemiological clue about the role of biomarker screening and the clinical prevention of CPL and CC.

## Ethics Statement

All patients involved in the study were required to read and sign the informed consent forms for both clinical epidemiological investigation and gene methylation testing prior to enrolment. Each participant was needed to be collected the peripheral venous blood and cervical epithelial exfoliated cells. The study was carried out in accordance with the recommendations of the Helsinki Declaration and the study protocol was approved by the ethics committee of The First Affiliated Hospital of Jinan University.

## Author Contributions

CJ and GY contributed to study conception and whole design. SH, RL, and XH wrote the present paper. SZ, LW, ZW, XZ, JW, YL, DL, YW, SD, XC, KZ, XD, ZZ, YH, XY, CZ, and BZ performed the experiments and carried out data collection. All authors approved the final version to be published.

## Funding

This work was supported in part by the Major Research Plan of the National Natural Science Foundation of China (91543132), National Natural Science Foundation of China (grant no: 81541070, 30901249, and 81101267), the Guangdong Natural Science Foundation (grant no: 2018A030313601, 10151063201000036, S2011010002526, and 2016A030313089), Guangdong Province Medical Research Foundation (grant no: A2014374, A2015310), and Project from Jinan university (grant no: 21612426, 21615426, JNUPHPM2016001, and JNUPHPM2016002).

## Conflict of Interest

The authors declare that the research was conducted in the absence of any commercial or financial relationships that could be construed as a potential conflict of interest.
