# Supplementary material for: Association Study Between Methylation in the Promoter Regions of cGAS, MAVS, and TRAF3 Genes and the Risk of Cervical Precancerous Lesions and Cervical Cancer in a Southern Chinese Population
Source: Front Genet. 2019 Nov 14;10:1123. doi: 10.3389/fgene.2019.01123 (PMC6868924; doi:10.3389/fgene.2019.01123)
Supplement: Supplementary file 1 [file DataSheet_1.docx]

Table S1 The primers of bisulfite sequencing polymerase chain reaction

| Gene | Primers | Amplicon size (bp) | Number of CpG sites |
| --- | --- | --- | --- |
| *cGAS* | Forward：AGATTAGGTTTTGTTGGGTTGTTTA | 143 | 9 |
|  | Reverse：CCTTTCCTAAAACTACAAAAATCTC |  |  |
| *MAVS* | Forward：GGTTTTTGTTTTAGAAGGGGAAG | 245 | 10 |
|  | Reverse：ACCCCCATAACCTAACATACAATTA |  |  |
| *TRAF3* | Forward：TTTGAGGTTTTTAAGGGGAGTTTAT | 125 | 5 |
|  | Reverse：CCCAACAAATACTTATTCTACAACCA |  |  |

Table S2 Association analysis of the promoter methylation status of *cGAS*/*MAVS*/*TRAF3* genes in each CpG site with the risk on CPL and CC

| CpG |  | CPL |  |  |  |  | CC |  |  |
| --- | --- | --- | --- | --- | --- | --- | --- | --- | --- |
| sites | OR (95% CI) | *P* | OR^a^ (95% CI) | *P*^a^ |  | OR (95% CI) | *P* | OR^a^ (95% CI) | *P*^a^ |
| *cGAS* |  |  |  |  |  |  |  |  |  |
| C1 | 2.55 (1.32-4.91) | **0.005** | 2.60 (1.34-5.02) | **0.005** |  | 3.21 (1.27-8.11) | **0.013** | 3.01 (1.14-7.97) | **0.027** |
| C2 | 2.52 (1.33-4.79) | **0.005** | 2.58 (1.35-4.92) | **0.004** |  | 3.06 (1.26-7.46) | **0.014** | 3.13 (1.22-8.08) | **0.018** |
| C3 | 2.17 (1.16-4.06) | **0.016** | 2.20 (1.17-4.14) | **0.014** |  | 0.66 (0.30-1.48) | 0.317 | 0.68 (0.28-1.62) | 0.379 |
| C4 | 1.42 (0.76-2.63) | 0.271 | 1.46 (0.78-2.73) | 0.233 |  | 0.66 (0.30-1.47) | 0.311 | 0.66 (0.28-1.57) | 0.351 |
| C5 | 2.81 (1.47-5.37) | **0.002** | 2.95 (1.53-5.68) | **0.001** |  | 0.75 (0.34-1.67) | 0.484 | 0.75 (0.31-1.81) | 0.526 |
| C6 | 2.81 (1.47-5.37) | **0.002** | 2.96 (1.53-5.71) | **0.001** |  | 0.96 (0.44-2.12) | 0.921 | 1.06 (0.44-2.54) | 0.895 |
| C7 | 1.96 (1.05-3.66) | **0.035** | 2.07 (1.10-3.89) | **0.024** |  | 0.72 (0.33-1.60) | 0.423 | 0.70 (0.29-1.71) | 0.436 |
| C8 | 2.69 (1.41-5.15) | **0.003** | 2.66 (1.39-5.11) | **0.003** |  | 0.43 (0.18-0.99) | **0.047** | 0.35 (0.14-0.89) | **0.027** |
| C9 | 1.25 (0.68-2.28) | 0.475 | 1.23 (0.67-2.25) | 0.513 |  | 0.37 (0.16-0.87) | **0.023** | 0.29 (0.11-0.75) | **0.011** |
| *MAVS* |  |  |  |  |  |  |  |  |  |
| M1 | 1.82 (0.98-3.36) | 0.058 | 1.83 (0.98-3.40) | 0.058 |  | 1.09 (0.49-2.39) | 0.840 | 0.83 (0.35-1.97) | 0.680 |
| M2 | 2.17 (1.16-4.06) | **0.016** | 2.20 (1.17-4.14) | **0.014** |  | 2.35 (1.01-5.45) | **0.047** | 2.14 (0.87-5.23) | 0.097 |
| M3 | 1.96 (1.05-3.66) | **0.035** | 1.97 (1.05-3.69) | **0.034** |  | 1.33 (0.60-2.95) | 0.484 | 1.26 (0.53-2.97) | 0.599 |
| M4 | 1.55 (0.84-2.87) | 0.158 | 1.63 (0.87-3.02) | 0.126 |  | 1.96 (0.86-4.48) | 0.111 | 2.44 (0.98-6.08) | 0.055 |
| M5 | 1.82 (0.98-3.36) | 0.058 | 1.97 (1.05-3.71) | **0.035** |  | 1.09 (0.49-2.39) | 0.84 | 1.51 (0.63-3.61) | 0.358 |
| M6 | 1.96 (1.05-3.66) | **0.035** | 1.94 (1.04-3.63) | **0.037** |  | 1.51 (0.68-3.37) | 0.317 | 1.38 (0.58-3.27) | 0.470 |
| M7 | 2.42 (1.27-4.60) | **0.007** | 2.51 (1.31-4.81) | **0.005** |  | 1.65 (0.73-3.71) | 0.230 | 1.53 (0.64-3.67) | 0.337 |
| M8 | 1.47 (0.80-2.70) | 0.216 | 1.50 (0.81-2.78) | 0.200 |  | 0.92 (0.42-2.03) | 0.840 | 0.73 (0.31-1.72) | 0.469 |
| M9 | 1.92 (1.04-3.58) | **0.039** | 1.94 (1.04-3.62) | **0.037** |  | 1.79 (0.79-4.03) | 0.162 | 1.45 (0.61-3.46) | 0.402 |
| M10 | 1.17 (0.64-2.13) | 0.617 | 1.18 (0.65-2.17) | 0.588 |  | 2.35 (1.01-5.45) | **0.047** | 2.28 (0.92-5.67) | 0.077 |
| *TRAF3* |  |  |  |  |  |  |  |  |  |
| T1 | 1.74 (0.94-3.23) | 0.078 | 1.85 (0.99-3.47) | 0.056 |  | 1.33 (0.60-2.95) | 0.484 | 1.91 (0.79-4.59) | 0.150 |
| T2 | 1.92 (1.04-3.58) | **0.039** | 1.87 (1.00-3.49) | **0.049** |  | 2.04 (0.90-4.66) | 0.090 | 2.07 (0.85-5.03) | 0.111 |
| T3 | 2.04 (1.09-3.81) | **0.025** | 2.07 (1.11-3.88) | **0.023** |  | 2.35 (1.01-5.45) | 0.047 | 3.19 (1.24-8.20) | 0.016 |
| T4 | 1.92 (1.04-3.58) | **0.039** | 1.89 (1.01-3.54) | **0.045** |  | 0.58 (0.26-1.31) | 0.194 | 0.59 (0.25-1.41) | 0.237 |
| T5 | 1.89 (1.02-3.51) | **0.043** | 1.86 (1.00-3.46) | **0.049** |  | 0.89 (0.40-1.96) | 0.766 | 1.09 (0.46-2.58) | 0.837 |

Statistically significant *p* values (*p*< 0.05) in bold font was based on logistic regression. ^a^Adjusted OR on age and number of pregnancy.

Table S3 Association analysis between hrHPV and the promoter methylation level of *cGAS*/*MAVS*/*TRAF3* genes among control, CPL and CC

| Methylation level | | HrHPV infection | | OR (95% CI) | *P* | OR^a^ (95% CI) | *P*^a^ |
| --- | --- | --- | --- | --- | --- | --- | --- |
|  |  | – | + |  |  |  |  |
| Control |  |  |  |  |  |  |  |
| *cGAS* | Low | 20 (40.82) | 29 (59.18) | 1 |  | 1 |  |
|  | High | 25 (52.08) | 23 (47.92) | 0.63 (0.28-1.42) | 0.267 | 0.65 (0.29-1.47) | 0.303 |
| *MAVS* | Low | 24 (48.98) | 25 (51.02) | 1 |  | 1 |  |
|  | High | 21 (43.75) | 27 (56.25) | 1.23 (0.56-2.75) | 0.606 | 1.20 (0.53-2.72) | 0.669 |
| *TRAF3* | Low | 23 (46.94) | 26 (53.06) | 1 |  | 1 |  |
|  | High | 22 (45.83) | 26 (54.17) | 1.05 (0.47-2.32) | 0.913 | 1.03 (0.46-2.30) | 0.947 |
| CPL |  |  |  |  |  |  |  |
| *cGAS* | Low | 6 (26.09) | 17 (73.91) | 1 |  | 1 |  |
|  | High | 13 (25.00) | 39 (75.00) | 1.06 (0.35-3.25) | 0.921 | 0.98 (0.31-3.16) | 0.976 |
| *MAVS* | Low | 9 (34.62) | 17 (65.38) | 1 |  | 1 |  |
|  | High | 10 (20.41) | 39 (79.59) | 2.07 (0.71-5.99) | 0.182 | 2.12 (0.71-6.35) | 0.178 |
| *TRAF3* | Low | 6 (19.35) | 25 (80.65) | 1 |  | 1 |  |
|  | High | 13 (29.55) | 31 (70.45) | 0.57 (0.19-1.72) | 0.321 | 0.47 (0.15-1.48) | 0.195 |
| CC |  |  |  |  |  |  |  |
| *cGAS* | Low | 3 (30.00) | 7 (70.00) | 1 |  | 1 |  |
|  | High | 0 (0.00) | 7 (100.00) | NA | NA | NA | NA |
| *MAVS* | Low | 1 (12.50) | 7 (87.50) | 1 |  | 1 |  |
|  | High | 2 (22.22) | 7 (77.78) | 0.50 (0.04-6.86) | 0.604 | 0.28 (0.01-5.36) | 0.394 |
| *TRAF3* | Low | 2 (25.00) | 6 (75.00) | 1 |  | 1 |  |
|  | High | 1 (11.11) | 8 (88.89) | 2.67 (0.19-36.76) | 0.464 | 1.91 (0.10-35.61) | 0.666 |

Low, *cGAS*≦31.87%, *MAVS*≦21.16%, *TRAF3*≦96.26%; High, *cGAS*>31.87%, *MAVS*>21.16%, *TRAF3*>96.26%. NA, not available, ^a^Adjusted OR on age and number of pregnancies

Table S4 Two-factor interaction of hrHPV with the promoter methylation level of *cGAS*/*MAVS*/*TRAF3* genes on CPL and CC

|  | RERI^a^ (95% CI) | | AP^a^ (95% CI) | | S^a^ (95% CI) | |
| --- | --- | --- | --- | --- | --- | --- |
| CPL |  |  |  |  |  |  |
| HrHPV & *cGAS* | 3.08 | (-0.82-6.98) | **0.54*** | **(0.13-0.95)** | 2.87 | (0.637-12.9) |
| HrHPV & *MAVS* | 1.89 | (-0.54-4.33) | **0.50*** | **(0.01-0.99)** | 3.14 | (0.326-30.22) |
| HrHPV & *TRAF3* | -0.56 | (-4.36-3.24) | -0.13 | (-0.98-0.73) | 0.86 | (0.332-2.24) |
| CC |  |  |  |  |  |  |
| HrHPV & *cGAS* | 0.94 | (-1.28-3.15) | 0.58 | (-0.69-1.85) | -2.00 | NA |
| HrHPV & *MAVS* | -0.28 | (-6.57-6.02) | -0.07 | (-1.67-1.53) | 0.91 | (0.128-6.54) |
| HrHPV & *TRAF3* | 1.95 | (-1.84-5.75) | 0.61 | (-0.28-1.51) | 9.05 | (0-23823273.65) |

S: synergy index, AP: attributable proportion due to interaction, RERI: relative excess risk due to interaction, 95% CI: 95% confidence interval. *, statistically significant. NA, not available.

Table S5 The interaction analysis of CPL risk estimates between hrHPV and *cGAS* promoter methylation level

| HrHPV | *cGAS*  Methylation level | OR^a^ (95%CI) | *P*^a^ |
| --- | --- | --- | --- |
| - | Low | 1 (Reference) |  |
| + | Low | 1.64 (0.62-4.37) | 0.319 |
| - | High | 1.94 (0.65-5.83) | 0.237 |
| + | High | 7.70 (2.84-20.88) | **< 0.001*** |

The combination of hrHPV-negative and cGAS promoter methylation levels≦31.87% is used for reference group. Low, *cGAS*≦31.87%; High, *cGAS*>31.87%; *, *P* < 0.001, adjusted OR on age and number of pregnancies.

Table S6 The interaction analysis of CPL risk estimates between hrHPV and *MAVS* promoter methylation level

| HrHPV | *MAVS*  Methylation level | OR^a^ (95%CI) | *P*^a^ |
| --- | --- | --- | --- |
| - | Low | 1 (Reference) |  |
| + | Low | 1.64 (0.63-4.25) | 0.307 |
| - | High | 1.39 (0.46-4.19) | 0.554 |
| + | High | 4.33 (1.78-10.52) | **0.001*** |

The combination of hrHPV-negative and *MAVS* promoter methylation levels≦21.16% is used for the reference group. Low, *MAVS*≦21.16%; High, *MAVS*>21.16%; *, *P* = 0.001

Table S7 MDR analysis of multi-factor interaction for CPL and CC

| Model | TBA (%) | CVC | *P* |
| --- | --- | --- | --- |
| CPL |  |  |  |
| HrHPV | 51.01 | 6/10 | 0.930 |
| HrHPV, *cGAS*promoter methylation level | 59.57 | 8/10 | 0.416 |
| HrHPV, *cGAS*promoter methylation level, age at first intercourse | 51.74 | 4/10 | 0.882 |
| HrHPV, *cGAS*promoter methylation level, age at first intercourse, number of abortions | 50.96 | 4/10 | 0.937 |
| CC |  |  |  |
| HrHPV | 74.24 | 10/10 | 0.021 |
| HrHPV, number of pregnancies | 73.66 | 8/10 | 0.060 |
| HrHPV, *cGAS*promoter methylation level, age at first intercourse | 59.84 | 7/10 | 0.503 |
| HrHPV, *cGAS* promoter methylation level, age at first intercourse, number of abortions | 65.93 | 6/10 | 0.279 |

TBA, Testing Balanced Accuracy; CVC, cross-validation consistency.
